# The Association between GP Consultations for Non-Specific Physical Symptoms in Children and Parents: A Case-Control Study

**DOI:** 10.1371/journal.pone.0108039

**Published:** 2014-09-24

**Authors:** Mujahed Shraim, Milisa Blagojevic-Bucknall, Christian D. Mallen, Kate M. Dunn

**Affiliations:** 1 Arthritis Research UK Primary Care Centre, Keele University, Keele, United Kingdom; 2 Work Environment Department, University of Massachusetts Lowell, Lowell, Massachusetts, United States of America; 3 Center for Disability Research, Liberty Mutual Research Institute for Safety, Hopkinton, Massachusetts, United States of America; Université Paris Descartes; AP-HP, Groupe Hospitalier Cochin-Saint-Vincent-de-Paul, France

## Abstract

**Background:**

Non-specific physical symptoms (NSPS) such as abdominal pain, headache and musculoskeletal pain are widespread in the community, and are common reasons for visiting a general practitioner (GP). Causes of NSPS are multifactorial, but may include parental influences.

**Objective:**

To investigate associations between GP consultations for NSPS in parents and their children.

**Methods:**

Matched case-control study using GP consultation data from 12 GP practices in the United Kingdom. Participants were 1328 children who consulted a GP for NSPS in 2009 (cases), 3980 controls who consulted a GP in 2009 but not for NSPS, plus parents of cases and controls (n = 8354). Primary outcome measure: child consultation status for NSPS.

**Results:**

Maternal consultation for NSPS was associated with significantly increased odds of their child consulting for NSPS (odds ratio (OR) 1.51, 95% confidence intervals (CI) 1.33, 1.73); there was no significant association with paternal consultations (OR 0.87, 95% CI 0.71, 1.08). Each additional maternal consultation for NSPS was associated with an increase in the rate ratio for number of consultations for NSPS in the child by 1.03 (95% CI 1.01, 1.05). This overall association was clearest in maternal-child consultations for painful NSPS and for specific bodily systems including gastrointestinal, musculoskeletal and neurologic symptoms.

**Conclusions:**

Maternal GP consultation for NSPS is associated with increased odds of GP consultations for NSPS in children. This study included a large sample of children and parents and used medical records data which is not subject to recall bias. However, analysis was based on medical records, thus the presence of NSPS not leading to consultations is unknown. Medical practitioners managing children with NSPS need to be aware of this association.

## Introduction

Non-specific physical symptoms (NSPS), such as musculoskeletal pain, abdominal pain, and headache are common in children [1], [2]. Annually, about one third of children consult a general practitioner (GP) for NSPS [3], [4]. NSPS among children are associated with functional impairment and negative impact on quality of life [5], [6]. This represents a significant burden on health care services through frequent general practitioner (GP) consultations, diagnostic testing, and referrals to secondary care services [7]–[9]. The causes of NSPS in children are yet to be fully explained, but are likely to be multifactorial, including genetic and psychosocial factors, such as parental influence on childhood illness and health-seeking behavior [3], [10], [11].

A recent systematic review found evidence of an association between GP consultations for NSPS in parents and children, but its findings were limited by the methodology used in the empirical studies, including the use of cross-sectional designs, reliance on self-reported data by either parents or children, and including children from specific age groups only [12]. Further research using documented GP consultation data may provide more precise information about the impact of parental NSPS on the child's health and GP consulting behavior for NSPS.

The objective of this study was to investigate the association between GP consultations for NSPS in parents and their children, assess whether this is different for mothers and fathers, and identify gradients of association for any observed associations.

## Methods

### Ethics statement

Ethical approval for the Consultations in Primary Care Archive (CiPCA) database was given by the North Staffordshire and Staffordshire Research Ethics Committees (UK), who gave permission to download and store anonymised medical record information for research use from participating general practices. All general practices participating in CiPCA inform their patient populations that their anonymised records will be used in this way and all patients are offered the opportunity to withdraw their records from inclusion in CiPCA.

### Study design and setting

This was a matched case-control study of children and their parents registered with UK GP practices. The setting was GP practices contributing to CiPCA, a primary care research database containing all recorded primary care consultations occurring at a subset of GP practices in Staffordshire, UK. In 2007, 12 GP practices contributed to the CiPCA database. The total population registered with these practices at mid-year 2009 was 104,911. CiPCA is a high quality, anonymized, and validated database, and the contributing practices have regular cycles of training, assessment and feedback with respect to the quality of coded clinical data [13]. Data from CiPCA on the annual consultation prevalence for musculoskeletal conditions are comparable to data from larger national primary care databases [14].

### Participants

Eligible participants were children and their parents registered with CiPCA practices between January 2007 and December 2009. We randomly selected only one child per household for inclusion in the study, because the main exposure of interest was parental consultation for NSPS, which would be the same for siblings; this thereby avoids over representing families with more than one child. Cases were defined as children, aged 2 to 16 years in 2009, who had at least one recorded GP consultation for NSPS between 1 January and 31 December 2009 (inclusive). We used the same criteria to define controls except that controls had at least one GP consultation, but not for NSPS.

We matched controls to cases on sex, maternal age group, and GP practice. We included between one to four controls per case (depending on the availability of suitable controls). If more than four controls per case were available, four were randomly selected. The sample size was calculated for 80% study power and 95% confidence using EpiCalc 2000 [15]. Sample size calculations showed that 535 children were required (107 cases and 428 controls) for each analysis (within mothers and children, and within fathers and children) to be able to detect an association between GP consultation for NSPS in parents and children with an odds ratio (OR) of 2, assuming the proportion exposed in the control group is 20%, and with 1∶4 ratio of cases to controls.

### Study variables

Outcome measures were the child's GP consultation status for NSPS and stratified by type of NSPS (painful and not-painful), different body systems, and single NSPS (see table 1), number of GP consultations for NSPS, and number of different NSPS consulted for.

**Table 1:** List of NSPS and their distribution in children and parents.

Non-specific physical symptoms	Case-children (n = 1328)	All mothers (n = 5308)	All fathers (n = 3058)
**Musculoskeletal symptoms**			
Pain in extremities	99 (7.5)	253 (4.8)	124 (4.1)
Back pain	59 (4.4)	460 (8.6)	237 (7.8)
Joint pain	209 (15.7)	627 (11.8)	148 (4.8)
Muscle soreness	5 (0.4)	15 (0.3)	7 (0.2)
**Gastrointestinal symptoms**			
Abdominal pain	346 (26.1)	547 (10.3)	130 (4.3)
Vomiting	146 (11.0)	39 (0.7)	13 (0.4)
Nausea	20 (1.5)	60 (1.1)	9 (0.3)
Bloating	3 (0.2)	37 (0.7)	6 (0.2)
Diarrhea	83 (6.3)	56 (1.1)	28 (0.9)
Constipation	132 (9.9)	59 (1.1)	10 (0.3)
Multiple food intolerance	0	0	0
Globus (lump in the throat)	0	7 (0.1)	0
Dysphagia (difficulty swallowing)	0	0	0
**Cardiopulmonary symptoms**			
Palpitations	9 (0.7)	17 (0.3)	34 (1.1)
Chest pain	62 (4.7)	211 (4.0)	119 (3.9)
Hyperventilation or Dyspnea	44 (3.3)	63 (1.2)	12 (0.4)
Hot or cold spells	1 (0.1)	17 (0.3)	4 (0.1)
**Urogenital symptoms**			
Pain during urination	1 (0.1)	0	0
Difficulty urinating (Dysuria)	77 (5.8)	4 (0.1)	3 (0.1)
Burning sensation in sexual organs or rectum	0	0	0
Dysmenorrhea (painful menstruation)	0	28 (0.5)	**
Metrorrhagia (irregular menstrual periods)	0	99 (1.9)	**
Menorrhagia (heavy menstrual bleeding)	0	210 (4.0)	**
Sexual indifference (decreased libido)*	*	16 (0.3)	16 (0.5)
Dyspareunia (pain during intercourse)*	*	26 (0.5)	**
**Neurologic symptoms**			
Dizziness/fainting (syncope)	22 (1.7)	168 (3.2)	43 (1.4)
Transient Amnesia (loss of memory)	0	4 (0.1)	1 (0.0)
Transient Aphonia (loss of voice)	0	0	0
Transient deafness	0	0	0
Transient Diplopia (double vision)	0	0	0
Transient blurred vision	1 (0.1)	4 (0.1)	0
Transient blindness	0	0	0
Transient seizure or convulsion	11 (0.8)	0	0
Transient Ataxia (trouble walking)	0	0	0
Transient Paresis (paralysis)	0	0	0
Headache	167 (12.6)	454 (8.6)	90 (2.9)
Paresthesia (numbness or tingling sensation)	4 (0.3)	58 (1.1)	33 (1.1)
Weakness in parts of the body	0	0	0
Heavy feelings in arms or legs	1 (0.1)	0	0
**General symptoms**			
Fatigue	68 (5.1)	225 (4.2)	58 (1.9)

NSPS =  non-specific physical symptoms; values are numbers (%); * =  Symptoms were excluded from analysis for children;** =  Not Applicable.

### Ascertainment of child GP consultations for NSPS

We identified GP consultations for NSPS by using a comprehensive list of standardized diagnostic Read codes referring to the list of NSPS presented in table 1. This list includes 33 NSPS from the diagnostic criteria in the third revision of Diagnostic and Statistical Manual of Mental Disorders for Somatization Disorder [16] and nine NSPS from the somatization factor of the Hopkins Symptom Checklist [17]. These NSPS were included in previous studies of Children's Somatization Inventory [18], [19] and other epidemiological studies investigating NSPS in children and adolescents [1], [20].

Read codes are a hierarchy of morbidity, symptoms and process codes that are used to record all electronic morbidity data in primary care in the UK [21]. We developed this list of Read codes by reviewing all Read codes referring to signs and symptoms in Read coding system. The full list of Read codes for included NSPS is available upon request from the authors. We classified GP consultations for NSPS as such if the reason for encounter was coded by the GP using any Read codes from our list. Also, one reviewer (MS) reviewed all free-text records for these consultations to ensure that the cause of symptoms (in the opinion of the GP) was not-specific: (a) not due to trauma or pregnancy (in mothers) (b) no recorded abnormalities on physical examination and/or diagnostic testing. If no information indicating (a) or (b) was available in the free-text records, we classified such consultations as consultations for NSPS.

### Assessment of exposure to parental consultations for NSPS

Within the CiPCA database, household identification codes were used to link household members, then date of birth was used to determine parents of included children. A mother/father of a randomly selected child within each household was defined as being a female/male aged 17 to 45 years at the birth of the child. All GP consultations made by parents between 1 January 2007 and 31 December 2008 were extracted from the CiPCA database. Maternal and paternal GP consultation status for NSPS between 2007 and 2008 were identified using the same method as described above for children. In children with GP consultation data on both parents, parental GP consultation status for NSPS was grouped into four categories. These comprised: both parents consulted for NSPS, only mother consulted for NSPS, only father consulted for NSPS, and neither parent consulted for NSPS. We also measured the total number of GP consultations for NSPS, and number of different NSPS in consulting parents during 2007-8. We also measured NSPS in parents sorted by painful and not-painful NSPS, body system, and single NSPS (table 1).

### Measurement of other independent variables

Besides variables used for case-control matching, we also obtained child's age, paternal age group, child birth order, household member count, index of multiple deprivation (IMD) 2007 scores for residential area level deprivation for included children, and parental history of anxiety or depressive disorders.

Children's age was split into tertiles (2–6, 7–11 and 12–16) and parents' age into quartiles (19–28, 29–39, 40–50 and 51–61 for mothers and 22–29, 30–40, 41–51 and 52–62 for fathers). Parents' age in 2009 rather than 2007 was used to reflect their age at the time of children's consultation. For example, mothers in the age group 19–28 years were two years younger at the time of their consultation in 2007.

Younger siblings for index children were defined as persons from the same household born after the index child, whereas older siblings were defined as persons from the same household and aged 16 or less at the birth of the index child. The birth order of the child was classified as “first” if the child had no siblings or if the child was the oldest child in the household (with no other household members' meeting the definition for a sibling).

The child household member count was dichotomized into households with three members or less and households with more than three households. Households with 13 or more members were excluded to prevent including families living in shared households. The IMD 2007 scores were constructed by the Department of Communities and Local Government, and conceptualized as a weighted area level aggregation of scores for seven domains of deprivation including income; employment; health deprivation and disability; education, skills and training; barriers to housing and services; crime; living environment [22]. IMD 2007 scores range from 0% to 100% where higher scores indicate greater deprivation [22]. IMD 2007 scores were also presented as quintiles with “1” representing the most affluent and “5” representing the most deprived. Maternal and paternal history of anxiety or depressive disorders status (yes, no) was identified by searching parental electronic primary care records between 2007 and 2008 using a list of symptom and diagnostic Read codes referring to anxiety and depressive disorders (this list is available on request from the authors).

### Statistical methods

Chi-squared tests and Mann-Whitney U tests were performed to test for significant baseline differences between cases and controls. We performed univariable analyses to examine crude associations between the independent variables and the child GP consultation status for NSPS using Cox regression. Effect size estimates were summarised using odds ratios (OR) with 95% confidence intervals (CI). We included all independent variables in the multivariable Cox regression to obtain adjusted associations between parental and child consultation for NSPS. In separate multivariable analyses, we also tested for any interaction effects between parental GP consultations for NSPS and other independent variables on the child GP consultation for NSPS.

In order to test for any potential gradient in associations, logistic regression was used to model effect of increasing number of paternal GP consultations for NSPS, and increasing number of different NSPS a parent consults for, on whether a child consults for NSPS. Similar analyses tested the relationship between consultation for multiple NSPS in parents and children. Furthermore, using Poisson regression we investigated whether there was a relationship between number of maternal consultations for NSPS and number of NSPS consultations in children.

In analyses for associations for painful and not-painful NSPS, body system, and single NSPS we adopted an unmatched case-control approach using logistic regression because relatively small number of cases have consulted for these symptoms categories. All analyses were carried out using SPSS (version 20.0) [23] and STATA (version 12) [24].

## Results

### Characteristics of cases and controls

5308 children were included (1328 cases and 3980 controls). The mothers of all children were identified. The fathers of 42.5% of cases and 42.7% of controls were unknown based on GP registration data, leaving 766 cases and 2292 controls with identifiable fathers. This may be because fathers were registered with other GP practices or were not registered with any practice at all, or because children were living with single-mothers. The baseline characteristics of all study participants are shown in table 2. Cases and controls differed significantly on all variables except for IMD 2007 scores and father history of anxiety or depressive disorders. Median age for cases was 10 years (Interquartile range 9) and 8 (8) years for controls (table 2). Maternal age was significantly different in cases and controls despite matching them on maternal age group. The most common single NSPS in children were abdominal pain, joint pain and headache. Abdominal pain, joint pain and back pain were the most common symptoms in parents (table 1).

**Table 2:** Baseline characteristics of children.

	Cases (n = 1328) n(%)^a^	Controls (n = 3980) n(%)^a^	*p*-value
**Child gender (Female)^b^**	739 (55.6)	2073 (52.1)	0.99
**Child age^c^**	10 (9)	8 (8)	<0.01
**Child age group^ b^**			
2–6 years	428 (32.2)	1688 (42.4)	
7–11 years	325 (24.5)	1114 (28.0)	
12–16 years	575 (43.3)	1178 (29.6)	
**Child birth order^ b^**			
First	63 (57.5)	2083 (52.3)	<0.01
Not first	565 (42.5)	1897 (47.7)	
**Household members**' **count**	4 (1)	4 (1)	0.02
**Household member count^ b^**			
≤3	691 (52.0)	1908 (47.9)	
>3	637 (48.0)	2072 (52.1)	
**IMD 2007 score^c^**	22.4 (22.9)	23.1 (22.9)	0.93
**IMD 2007quartiles^ b^**			
I	272 (20.5)	819 (20.6)	
II	262 (19.7)	785 (19.7)	
III	263 (19.8)	792 (19.9)	
IV	280 (21.1)	809 (20.3)	
V	244 (18.4)	759 (19.1)	
Missing IMD score **^b^**	7 (0.5)	16 (0.4)	
**Mother age^c^**	39 (11)	38 (9)	<0.01
**Mother age group^ b^**			
19–29 years	216 (16.3)	722 (18.1)	
30–40 years	604 (45.5)	1972 (49.5)	
41–51 years	475 (35.8)	1227 (30.8)	
52–62 years	33 (2.5)	59 (1.5)	
**Father age^c^**	41 (10)	40 (9)	<0.01
**Father age group^b^**			
22–32 years	101 (13.2)	378 (16.6)	
33–43 years	381 (49.9)	1241 (54.4)	
44–54 years	246 (32.2)	590 (25.9)	
55–65 years	36 (4.7)	73 (3.2)	
**Mother history of anxiety or depressive disorder 2007–2008^ b^**			
No	993 (74.8)	3188 (80.1)	<0.01
Yes	335 (25.2)	792 (19.9)	
**Father history of anxiety or depressive disorder 2007–2008^b,d^**			
No	699 (91.4)	2110 (92.4)	0.39
Yes	65 (8.5)	172 (7.5)	

a Percentages may not total 100 due to rounding; ^b^Number (%); ^c^Median (Interquartile range) Index of Multiple Deprivation 2007; ^d^564 (42.5%) of cases and 1698 (42.7%) of controls had no paternal records. Controls were matched to cases on sex, maternal age group, and GP practice.

### The association between GP consultations for NSPS in parents and children

Overall, 52% of cases and 41% of controls had a maternal history of consultation for NSPS, and 30% of cases and controls (with available paternal records) had paternal history of consultation for NSPS.

Initially, analyses included all children, irrespective of whether both parents were identified. Univariable analysis revealed that cases were more likely than controls to have a history of maternal GP consultation for NSPS (crude OR 1.55, 95% CI 1.37, 1.76), a finding which persisted in multivariable analysis (adjusted OR 1.51, 95% CI 1.33, 1.73) after adjustment for child age group, father age group, child birth order, household members' count, and maternal history of anxiety and depressive disorders. We found no significant association between GP consultation for NSPS in fathers and children (table 3). Subsequently, analyses included only those children who had both parents identified in the study sample (764 cases and 2183 controls).

**Table 3:** Associations between consultations for NSPS in either parant and children.

	Cases (n = 1328) n(%)	Controls (n = 3980) n(%)	Crude OR (95% CI)	Adjusted OR (95% CI)
**Maternal consultation history for NSPS 2007–2008**				
Yes	695 (52.3)	1642 (41.3)	1.55 (1.37, 1.76)	1.51 (1.33, 1.73)
No	633 (47.7)	2338 (58.7)	1	1
**Paternal consultation history for NSPS 2007–2008**				
Yes	228 (29.9)	692 (30.4)	0.89 (0.73, 1.09)	0.87 (0.71, 1.08)
No	536 (70.1)	1590 (69.6)	1	1

NSPS =  non-specific physical symptoms; OR =  odds ratio; CI =  confidence intervals; 564 (42.5%) of cases and 1698 (42.7%) of controls had no paternal records. ORs were adjusted for child age, father age, child birth order; household members' count, Index of Multiple Deprivation 2007 scores; maternal and paternal anxiety or depressive disorder.

We found that in instances where only a mother consulted for NSPS, the odds of a child having a GP consultation for NSPS was 1.38 (95% CI 1.14, 1.69) as compared to a child with neither parent consulting for NSPS. We found no significant association between GP consultations for NSPS in fathers and children (see Table 4). However, GP consultation for NSPS in both parents was associated with increased odds of child consulting for NSPS (adjusted OR 1.57, 95% CI 1.23, 2.01).

**Table 4:** Associations between consultations for NSPS in both parents and children.

	Cases (n = 764) n(%)	Controls (n = 2183) n(%)	Crude OR (95% CI)	Adjusted OR (95% CI)
**Both parents did not consult for NSPS**	276 (36.1)	989 (45.3)	1	1
**Only father consulted for NSPS**	78 (10.2)	335 (15.3)	0.82 (0.63, 1.08)	0.83 (0.63, 1.10)
**Only mother consulted for NSPS**	260 (34.0)	568 (26.0)	1.44 (1.20, 1.74)	1.38 (1.14, 1.69)
**Both parents consulted for NSPS**	150 (19.6)	291 (13.3)	1.63 (1.29, 2.06)	1.57 (1.23, 2.01)

NSPS =  non-specific physical symptoms; OR =  odds ratio; CI =  confidence intervals. ORs were adjusted for child age, father age, child birth order; household members' count, Index of Multiple Deprivation 2007 scores; maternal and paternal anxiety or depressive disorder.

No significant interaction effects were identified between parental consultation for NSPS and other independent variables on the child GP consultation status for NSPS.

### Gradients association between GP consultations for NSPS in parents and children

Analyses for gradients association were restricted to mother-child pairs because we found no significant associations between GP consultations for NSPS in fathers and children. Logistic regression analysis showed a significant increase in the odds of child consulting for NSPS with increasing number of GP consultations for NSPS and number of different NSPS consulted for in the mother (see table 5). Children whose mother consulted only once had odds of consulting almost 1.4 times higher than children whose mother didn't consult. These odds increased to 2.14 (95% CI 1.68, 2.72) for those children whose mother consulted more than three times.

**Table 5:** Associations between number of consultations for NSPS and number of different NSPS in mother and child consultation for NSPS.

NSPS	Cases n = 1328 n(%)	Controls n = 3980 n(%)	Crude OR (95% CI)	Adjusted OR (95% CI)
**Maternal number of consultations for NSPS**				
0	633 (47.7)	2338 (58.7)	1	1
1	300 (22.6)	809 (20.3)	1.37 (1.17, 1.61)	1.38 (1.17, 1.63)
2	156 (11.7)	406 (10.2)	1.41 (1.14, 1.73)	1.36 (1.10, 1.68)
3	96 (7.2)	198 (5)	1.77 (1.36, 2.31)	1.69 (1.28, 2.22)
>3	143 (10.8)	229 (5.8)	2.24 (1.78, 2.82)	2.14 (1.68, 2.72)
**Maternal number of different NSPS**				
0	663 (47.7)	2338 (58.7)	1	1
1–2	601 (45.3)	1494 (37.5)	1.47 (1.29, 1.68)	1.45 (1.27, 1.66)
>2	94 (7.1)	148 (3.7)	2.37 (1.79, 3.12)	2.24 (1.68, 2.99)

OR =  odds ratio; CI =  confidence intervals. ORs were adjusted for child age, child birth order; household members' count, Index of Multiple Deprivation 2007 scores; maternal anxiety or depressive disorder.

Restricting the analysis only to cases, Poisson regression showed that each additional one maternal GP consultation for NSPS was associated with increase in the rate ratio for number of consultations for NSPS in the child by 1.03 (95% CI 1.01, 1.05). Additionally, using logistic regression, we found that cases whose mothers consulted with multiple NSPS (two or more NSPS) had increased odds of consulting with multiple NSPS as compared to cases whose mothers did not consult for NSPS (adjusted OR 2.24, 95% CI 1.68, 2.99).

### The association between GP consultations for NSPS groups and single NSPS in parents and children

As shown in table 6, we found significant associations between consultations for painful NSPS in mothers and children (OR 1.49, 95% CI 1.28, 1.74), but not for not-painful NSPS (OR 1.11, 95% CI 0.88, 1.40). Logistic regression analysis showed significant associations between maternal and child consultations for musculoskeletal, gastrointestinal, and neurologic symptoms, but not for cardiopulmonary and urogenital symptoms (table 6). Additionally, the association between maternal and child consultations for single NSPS was statistically significant for abdominal pain, joint pain, extremities pain, headache, and vomiting (table 6)

**Table 6:** Associations between consultation for specific symptoms in mothers & children.

	NSPS in the child			
NSPS in the mother	Yes	No	Crude OR (95% CI)	Adjusted OR (95% CI)
**Painful NSPS**				
Yes	376	1441	1.53 (1.32, 1.77)	1.49 (1.28, 1.74)
No	509	2982	1	1
**Not-painful NSPS**				
Yes	111	843	1.21 (0.97, 1.50)	1.11 (0.88, 1.40)
No	429	3925	1	1
**Musculoskeletal NSPS**				
Yes	130	1128	1.53 (1.23, 1.90)	1.41 (1.22, 1.75)
No	284	3766	1	1
**Gastrointestinal NSPS**				
Yes	112	576	1.51 (1.21, 1.89)	1.40 (1.12, 1.76)
No	527	493	1	1
**Neurological NSPS**				
Yes	44	558	1.92 (1.36, 2.69)	2.10 (1.46, 3.02)
No	186	4520	1	1
**Cardiopulmonary NSPS**				
Yes	5	210	1.62 (0.65, 4.04)	1.27 (0.50, 3.25)
No	74	5019	1	1
**Urogenital NSPS**				
Yes	5	352	0.96 (0.39, 2.40)	0.90 (0.36, 2.26)
No	72	4879	1	1
**Abdominal pain**				
Yes	59	488	1.89 (1.40, 2.53)	1.81 (1.34, 2.44)
No	287	4474	1	1
**Joint pain**				
Yes	37	590	1.64 (1.14, 2.37)	1.55 (1.07, 2.25)
No	172	4509	1	1
**Headache**				
Yes	23	431	1.75 (1.11, 2.74)	2.02 (1.26, 3.24)
No	144	4710	1	1
**Vomiting**				
Yes	6	33	6.67 (2.75, 16.15)	4.98 (2.00, 12.40)
No	140	5129	1	1
**Constipation**				
Yes	1	58	0.67 (0.09, 4.90)	0.60 (0.08, 4.40)
No	131	5118	1	1
**Pain in extremities**				
Yes	10	243	2.30 (1.18, 4.47)	2.12 (1.08, 4.16)
No	89	4966	1	1
**Diarrhea**				
Yes	2	54	2.36 (0.57, 9.86)	2.17 (0.51, 9.36)
No	81	5171	1	1
**Fatigue**				
Yes	2	223	0.68 (0.17, 2.80)	0.70 (0.17, 2.89)
No	66	5017	1	1
**Back pain**				
Yes	8	452	1.67 (0.79, 3.53)	1.42 (0.66, 3.05)
No	51	4797	1	1
**Fainting/dizziness**				
Yes	1	167	0.54 (0.08, 3.95)	0.52 (0.07, 3.86)
No	56	5084	1	1

OR =  odds ratio; CI =  confidence intervals. ORs were adjusted for child age, child sex, mother age, child birth order; household members' count, Index of Multiple Deprivation 2007 scores; maternal anxiety or depressive disorder, and GP practice.

## Discussion

This study found statistically significant associations between GP consultations for NSPS in mothers (but not fathers) and their children. The association between parental and child consultation was stronger when both parents consulted for NSPS. We also found a statistically significant gradient of association between the number of GP consultations for NSPS and the number of NSPS in mothers and children. Additionally, we found significant associations between maternal and child consultation for painful symptoms, specific body systems, and single NSPS.

The observed associations between GP consultations for NSPS in mothers and children are consistent with the findings of a systematic review which found evidence of an association between GP consultations for NSPS in parents and children [12]. However, that review found only eight studies, and the majority of studies used self-reported data by either parents or children and used cross-sectional designs. One study reported that the associations between GP consultations for abdominal pain or headache were greater for mother-child pairs than father-child pairs [3]. Another study found maternal GP consultations NSPS more predictive of the child consultations for gastrointestinal NSPS than those of fathers [25].

With respect to the significant association between maternal and child consultation for painful NSPS, but not for not-painful symptoms, it may be that consulting behavior is influenced by level of pain severity and perceived seriousness of the symptoms [26], [27]. For example, parents may perceive painful NSPS in themselves and in their children as more serious than not-painful NSPS, which influences their GP consultation rates for these NSPS.

One possible explanation for these associations is an inherited genetic predisposition to NSPS. There is some evidence that genetic effects contribute to the onset of some NSPS and syndromes, such as headache and irritable bowel syndrome [28], [29]. However, it is seems unlikely for genetic factors to fully explain this, because only maternal (and not paternal) consultations for NSPS were associated with the child consulting for NSPS. Another explanation is shared exposure of family members to certain social and environmental factors (e.g. stressful events, lack of social support, socioeconomic circumstances, and poor family functioning) which have been found to be associated with greater reporting of NSPS [30], [31] and GP consultations for NSPS in parents and children [7]. One plausible explanation is the childhood social learning of illness behavior, which has been hypothesised to play an important role in the development of illness and healthcare seeking behavior among children [32]–[34]. A number of studies have suggested that parental responses and attitudes toward the child illness (reinforcement) and parental coping mechanisms with their own illness (role modelling) may influence symptom frequency, disability days, and healthcare consultations in their children [35], [36]. We would expect social learning and role modeling to increase with child's age. However, we found no significant interaction effects between the child age group (or other independent variables) and maternal consultations for NSPS on the child GP consultation status for NSPS. This suggests that the maternal effect on child consultations for NSPS through “reinforcement” is present at all ages and represent a more important influence.

### Strengths and limitations

The main limitations of the previous few studies that examined the association between GP consultations for NSPS in parents and children were the use of self-reported data which is prone to recall bias, including children from specific age groups, and the use of cross-sectional designs that are unable to distinguish the direction of associations. Strengths of our study therefore include the fact that we used documented GP consultations, which are more precise source of information on attendance in primary care than relying on self-reported data by children or their parents that may be prone to recall bias [37]. Additionally, we included a large sample (n = 5308) and relied on documented GP attendance from 12 practices which increases the internal and external validity of our findings. Also, as described in the methods section, CiPCA is a high quality validated database, which enhances the validity of our findings. For example, 97% of all GP consultations that occurred in the CiPCA practices in 2006 were given a morbidity code [38]. Another important strength is that parental consultation status for NSPS was ascertained using parental consultations data in the two years preceding the child consultation, which provides a clear temporal relationship. Additionally, the observed associations were supported by evidence of gradients of association.

One limitation is the potential for diagnostic misclassification, which is a common problem in primary care [39]. However, diagnostic misclassification is unlikely to completely explain the associations found in this study due to the high quality of coded clinical data within CiPCA practices. Additionally, the current classification system used in primary care allows for coding definitive diagnoses (e.g. urinary tract infection) as well as symptoms' diagnoses (e.g. abdominal pain) when a definitive diagnosis is not established, which reduces the potential for diagnostic misclassification. One potential limitation is the lack of blinding of GPs to symptoms in parents and children at time of consultation. However, we believe that this is minimal and not systematic because it does not explain differences in associations between mothers and fathers, and that our findings were based on association between parents' consultations in 2007–2008 and the child's consultations in 2009. Another limitation is that, based on GP practice registered population data, we were not able to examine whether our findings are different for children living or not living in the same house as their mother and father. Also, paternal consultations status for NSPS was unknown for 43% of children. However, this was distributed equally between cases and controls. Additionally, it is unlikely that the lack of significant associations between GP consultations for NSPS in fathers and children is entirely attributed to low study power as the numbers of children with paternal consultation data were more than those needed based on sample size calculations. Although we matched controls and cases on maternal age group, they differed significantly on maternal age. However, it is unlikely for this to lead to significant changes in our ORs estimates because we also found statistically significant associations for single NSPS in mother and children after adjustment for maternal age in unmatched analyses.

Although this study set out to examine the association between consultation patterns for NSPS in parents and children, GP consultation data only provides a measure of health problems for which practice registered populations have consulted. Thus, the presence of other NSPS which did not result in GP consultation remains unknown.

### Generalizability

In the UK over 97% of the population is registered with a GP practice, which usually provides first point access to non-emergency healthcare [40]. We used a large population-based sample of families registered with GP practices from Staffordshire. This area is more deprived than England as a whole, but we found no significant associations between child GP consultation for NSPS and area level deprivation in either univariable or multivariable analyses. Although the healthcare setting in UK may be different from other healthcare settings, similar associations for specific symptoms between parents and children were reported in different countries [3], [25], [41].

### Implications for clinical practice and future research

These findings indicate that GPs and other medical practitioners managing children with NSPS in secondary care settings should be aware of the association between consultations for NSPS in parents and children, as such insights might direct medical practitioners toward alternative management approaches. For example, a recent randomized controlled trial has demonstrated that cognitive behavioral therapy (CBT) targeting children's coping responses to recurrent abdominal pain and parents' responses to pain in their children was associated with significant reduction in pain and gastrointestinal symptoms severity in children at six month follow-up [42]. It is not clear whether CBT had an impact on the consultation behavior of children for NSPS. There is some evidence from literature on adults that CBT, pharmacological therapy, and aerobic exercise for patients presenting with NSPS in primary care and general outpatient clinics are effective in reducing frequency of symptoms, the number of consultations, and related psychological distress [43]–[46]. Potentially, such interventions for parents could impact on the illness and healthcare seeking behavior of both parents and their children, but no studies exist to confirm or refute this. This provides a rationale for future research to focus on development of appropriate clinical guidelines on management of parents and children with consultations for NSPS. Additionally, more prospective and qualitative research is required to fully explain the exact mechanisms underlying the association between GP consultations for NSPS in mothers and children. Such research may shed light on interventions that may help in preventing the development or recurrence of consultations for NSPS in children.

### Conclusions

This study suggests that exposure to maternal GP consultations for NSPS is a significant risk factor for similar consultations in the child. This finding was strengthened by evidence of gradients of association between number of GP consultations for NSPS and number of NSPS in mothers and children as well as associations for specific type symptom groups and single NSPS especially painful NSPS. This study adds further evidence that children may learn their illness and consultation behavior from their mothers, and that recurrent consultation for NSPS in children should be viewed within a family context.
